# *Epichloë* seed transmission efficiency is influenced by plant defense response mechanisms

**DOI:** 10.3389/fpls.2022.1025698

**Published:** 2022-10-21

**Authors:** Wei Zhang, Natasha T. Forester, Christina D. Moon, Paul H. Maclean, Milan Gagic, Sai Krishna Arojju, Stuart D. Card, Cory Matthew, Richard D. Johnson, Linda J. Johnson, Marty J. Faville, Christine R. Voisey

**Affiliations:** ^1^ Grasslands Research Centre, AgResearch Limited, Palmerston North, New Zealand; ^2^ School of Agriculture and Environment, Massey University, Palmerston North, New Zealand

**Keywords:** *Epichloë*, fungal endophyte, genotyping-by-sequencing, perennial ryegrass, plant disease resistance, transcriptome, vertical transmission

## Abstract

Asexual *Epichloë* are endophytic fungi that form mutualistic symbioses with cool-season grasses, conferring to their hosts protection against biotic and abiotic stresses. Symbioses are maintained between grass generations as hyphae are vertically transmitted from parent to progeny plants through seed. However, endophyte transmission to the seed is an imperfect process where not all seeds become infected. The mechanisms underpinning the varying efficiencies of seed transmission are poorly understood. Host gene expression in response to *Epichloë* sp. LpTG-3 strain AR37 was examined within inflorescence primordia and ovaries of high and low endophyte transmission genotypes within a single population of perennial ryegrass. A genome-wide association study was conducted to identify population-level single nucleotide polymorphisms (SNPs) and associated genes correlated with vertical transmission efficiency. For low transmitters of AR37, upregulation of perennial ryegrass receptor-like kinases and resistance genes, typically associated with phytopathogen detection, comprised the largest group of differentially expressed genes (DEGs) in both inflorescence primordia and ovaries. DEGs involved in signaling and plant defense responses, such as cell wall modification, secondary metabolism, and reactive oxygen activities were also abundant. Transmission-associated SNPs were associated with genes for which gene ontology analysis identified “response to fungus” as the most significantly enriched term. Moreover, endophyte biomass as measured by quantitative PCR of *Epichloë* non-ribosomal peptide synthetase genes, was significantly lower in reproductive tissues of low-transmission hosts compared to high-transmission hosts. Endophyte seed-transmission efficiency appears to be influenced primarily by plant defense responses which reduce endophyte colonization of host reproductive tissues.

## Introduction

Many members of the subfamily Pooideae (within the family Poaceae), also known as the cool-season grasses, are hosts to *Epichloë* (family Clavicipitaceae) fungal endophytes (Selosse and Schardl, 2007). The grass hosts and endophytes form intimate symbioses that span a range of ecological relationships from mutualistic to antagonistic (Schardl et al., 1997). Within mutualistic endophyte-grass associations, *Epichloë* hyphae grow asymptomatically within the grass host, residing in the intercellular spaces of aerial tissues where they derive nutrition from within the apoplast (White Jr. et al., 1991; Christensen and Voisey, 2009). In turn, the endophytes in general can confer a range of benefits to the host, most importantly protection against mammalian and insect herbivores through the production of fungal bioactive secondary metabolites (Johnson et al., 2013; Johnson et al., 2021). These traits confer significant fitness advantages to the host, compared to their endophyte-free counterparts, and may result in selection for endophyte infection in grass populations under stressful conditions. For example, natural grasslands in Mediterranean regions that experience low water availability and hot summer temperatures can exhibit high proportions of endophyte infection that presumably arise because the endophyte confers a selective advantage under these conditions (Lewis et al., 1997; Saikkonen et al., 2000).

Perennial ryegrass (*Lolium perenne*) is an important agricultural forage species in temperate climates, and elite cultivars have been inoculated with selected, characterized strains of asexual *Epichloë* (Johnson et al., 2013; Johnson et al., 2021). These fungal strains have been successfully marketed in seed for almost three decades for their insect deterrence and wildlife management traits, with minimal toxicity to grazing livestock (Caradus et al., 2021). In New Zealand, the presence of *Epichloë* is essential for pasture persistence in many regions that experience high insect pest pressure (Johnson et al., 2013). In particular, the widely-adopted pasture endophyte strain, AR37, confers strong feeding deterrence against a number of insect species, with few detrimental effects to grazing animals (Johnson et al., 2021). However, the transmission of AR37 from the parent plant to the seed is highly variable (Gagic et al., 2018), which can compromise the quality of commercial seed products (Johnson et al., 2013; Johnson et al., 2019).

Asexual *Epichloë* species colonize the apoplastic spaces within the grass host’s aerial tissues and are clonally transmitted to seed *via* vertical transmission. The growth of asexual *Epichloë* spp. is highly synchronized with that of its host grasses (Tan et al., 2001; Christensen et al., 2008). In vegetative tissues, hyphal tip growth occurs between meristematic cells of the shoot apex and organ primordia while hyphal compartments that are attached to extending host cells co-migrate in synchrony with cell files of maturing leaves (Christensen et al., 2008). This co-migration process also takes place throughout host floral (inflorescence) development to ovules within florets of the spikelets and to the embryos of developing host seed of the next generation of seedlings (Liu et al., 2017; Zhang et al., 2017). Vertical transmission of endophyte to the seed is therefore dependent on infection of each host inflorescence primordium (IP) and ultimately of individual ovaries (OV) (Gundel et al., 2011; Gagic et al., 2018).

Insights into the mechanisms by which endophytes and their grass hosts interact have been greatly enriched through transcriptomic analyses of naturally-evolved and artificially-established associations, and these studies show varied host responses (Dupont et al., 2015; Dinkins et al., 2017; Wang et al., 2021). Naturally-evolved associations exhibit a relatively small number of alterations in gene expression during symbiosis, such as tall fescue in natural association with *E. coenophiala* (Dinkins et al., 2017). In contrast, analysis of an artificial association between a clonal line of perennial ryegrass cv. Nui and *E. festucae* var. *lolii* strain Lp19 (originally also from *L. perenne*) revealed mild defense responses in the host that included an increase in the expression of genes associated with hormone biosynthesis and perception, stress and pathogen resistance, and decreased expression of photosynthesis-related genes (Schmid et al., 2017). It was suggested that this response may help prime host plant immunity to pathogens and other biotic stresses.

In a previous study we tested the hypothesis that both host genetics and environment influence the transmission of *Epichloë* in seeds. We found that host genotype is more important than the environments examined in influencing AR37 seed infection, and genotypes that had been selected for higher transmission rates supported higher AR37 biomass and retained endophyte infection over longer periods of time (Gagic et al., 2018). We therefore hypothesized that host/endophyte associations with low AR37 transmission to seed may be less compatible, limiting hyphal colonization of floral tissues and reducing infection of the embryo in the developing seed. While many studies have compared the transcriptomes of endophyte-infected with endophyte-free hosts (Dupont et al., 2015; Dinkins et al., 2017; Schmid et al., 2017), few have examined the impact of host grass genotype in response to endophyte infection with the same fungal strain. This current study aimed to determine the molecular processes underlying the contrasting AR37 seed transmission outcomes in perennial ryegrass. Using two independent approaches, we examined the transcriptomes of reproductive tissues (IP and OV) from high-transmission (HT) and low-transmission (LT) host genotypes symbiotic with AR37, and we also undertook population level analyses of genes associated with SNPs that were the most predictive of endophyte transmission efficiency. Together these data provide new insights into host mechanisms underpinning successful transmission of *Epichloë* spp. into the next generation of plants.

## Materials and methods

### Plant materials and tissue sampling

**Figure 1:**
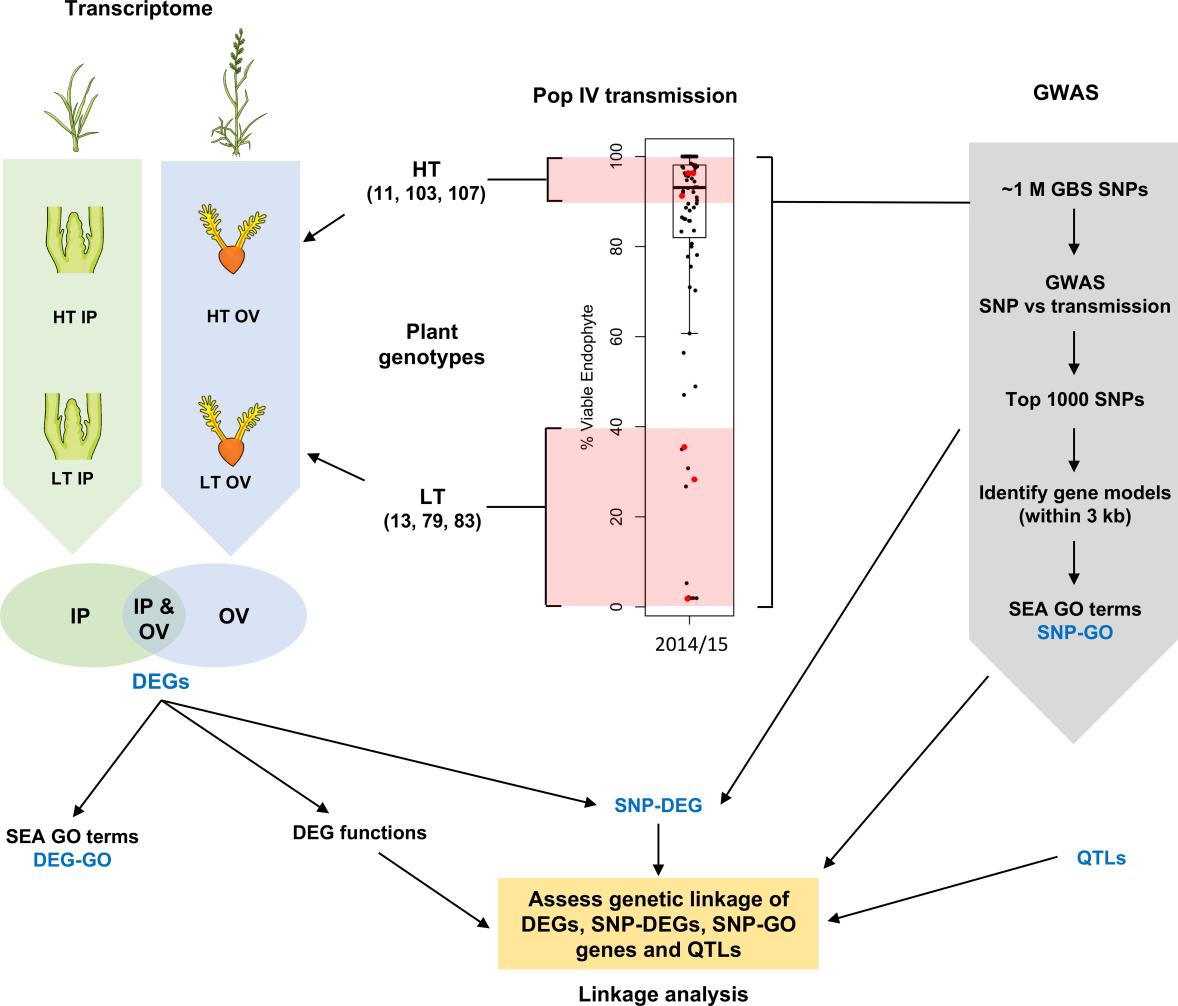
Schematic overview of this study. Inflorescence primordium (IP) and ovary (OV) tissues from three high-transmission (HT) and low-transmission (LT) genotypes within a breeding population (red dots) were chosen for transcriptome analysis, and compared to genes associated with informative single nucleotide polymorphisms (SNPs); the latter determined by genome-wide association studies (GWAS). DEG, differentially-expressed gene; GBS, genotyping by sequencing; GO, gene ontology; SEA, singular enrichment analysis; QTL, quantitative trait loci. The boxplot uses data published in Figure 5 (Population IV, 2014 at the Grasslands site, New Zealand) of Gagic et al., 2018 and is reproduced here under the Creative Commons Attribution License (CC BY).

A breeding population of perennial ryegrass (*Lolium perenne*) that was artificially inoculated with *Epichloë* sp. LpTG-3 (*Lolium perenne* taxonomic group 3) (Hettiarachchige et al., 2015) strain AR37 was maintained at the AgResearch Grasslands Research Centre, New Zealand (previously described by Faville et al. (2018)). This population, number IV, exhibited a range of viable endophyte transmission frequencies to seed. Six plant genotypes from this population with high frequencies of tiller infection, and that displayed consistently high seed transmission efficiency (>75% viable endophyte; plant IDs 11, 103, 107) or consistently low seed transmission efficiency (<50% viable endophyte; plant IDs 13, 79, 83) over the 2013/14, 2014/15 seed production seasons [previously described in Gagic et al. (2018)], were selected for this study (**Figure 1**). The endophyte infection status of these plant genotypes was further assessed four years later during the 2019/20 seed production season using an established tissue-print immunoblot assay technique as previously described by Gagic et al. (2018). The transmission of viable endophyte to seedlings (%) was determined by tissue-print immunoblot testing of 120 seedlings derived from each of the six plant genotypes.

In May 2015, ten endophyte-infected tillers of each plant genotype were propagated in triplicate in 1 liter of a bark-based potting mix (Gagic et al., 2018). The endophyte infection status of each tiller was determined by staining leaf sheath tissues with aniline blue and observing by light microscopy (Bacon et al., 1977). IP and OV samples were harvested in November and December of 2015, respectively. Harvest was conducted over a one-week period for each tissue type at solar noon (± 1 hr) to reduce transcriptome variability due to diurnal variation. Several IP samples were harvested and their developmental stages determined according to Zadoks et al. (1974). The endophyte infection status of each tiller from which each IP was collected was confirmed by microscopy. IP tissues at the stage equivalent to growth stage 31-32 (Zadoks et al., 1974) were chosen for RNA extraction, with seven infected IP samples per plant genotype pooled together to obtain sufficient RNA for downstream analyses.

**Figure 2:**
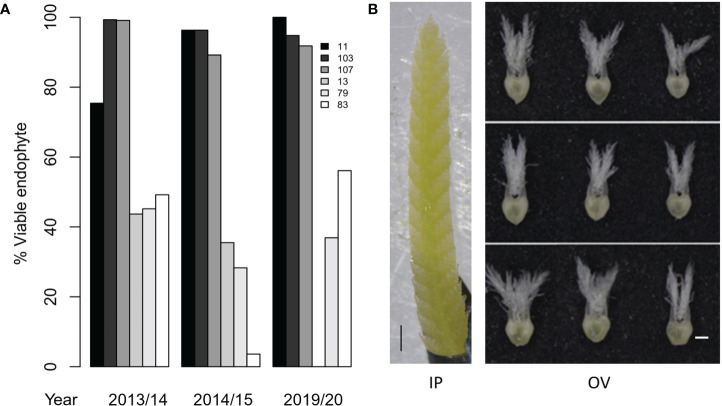
Phenotypes of perennial ryegrass genotypes with high and low AR37 transmission in Population IV. **(A)** Endophyte transmission into seedlings for the six AR37-infected perennial ryegrass genotypes examined in this study in 2013/14, 2014/15 and 2019/20. Seed was not produced by genotype 13 in 2019/20, thus transmission data could not be obtained. **(B)** Developmental stages of ryegrass an inflorescence primordium (IP) and ovary (OV) tissues used for transcriptome analysis. An IP is depicted on the left, and OV tissues from the bottom, middle and top of spikelets are shown on the right. Scale bars = 500 µm.

OV samples from an individual plant genotype were collected when the emergence of the inflorescence was completed (growth stage 58-59) (Zadoks et al., 1974) from three reproductive tillers with a similar emergence date (± 2 days). Nine OVs were collected per tiller, three from the top, three from the middle and three from the base of each spikelet at (third from) top, center and (third from) bottom of the spike (**Figure 2**), these being the bottom three OVs from the bottom three florets of each spikelet. The rachilla of each floret that the OV was harvested from was blotted using tissue-print immunoblot to confirm endophyte infection. In total, 27 infected OVs from each plant genotype were pooled for RNA extraction.

### RNA extraction

Each IP sample or OV sample was powdered in liquid nitrogen using a micropestle in a 1.5 mL microcentrifuge tube. The RNA extraction method used for IP was a combination of an organic extraction using TRIzol™ reagent (Thermo Fisher Scientific Inc., USA) followed by further purification of 200 µL of the TRIzol/chloroform aqueous phase with an RNeasy MinElute Cleanup Kit (Qiagen, USA). RNA was extracted from OV samples using a method suitable for carbohydrate rich samples (Wang et al., 2012), which combined an extraction with sodium dodecyl sulfate and TRIzol/chloroform followed by the purification with RNeasy MinElute Cleanup Kit. Each RNA sample was eluted in 15 µL nuclease-free water and stored at –80°C. RNA quality was determined using an Agilent 2100 Bioanalyzer with RNA 6000 Nano LabChip (Agilent Technologies, USA) where RNA integrity numbers >8 were obtained.

### Transcriptome analyses

Sequencing libraries were prepared from the IP and OV RNA samples using the TruSeq stranded mRNA sample preparation kit (Illumina Inc., USA). The indexed libraries for the IP samples were pooled and sequenced on two lanes of an Illumina HiSeq 2500 instrument at New Zealand Genomics Ltd., New Zealand, while the OV samples were sequenced using the Illumina HiSeq™ 4000 platform (BGI, Hong Kong). Approximately 100 million read pairs of raw sequence data was generated per library.

Trimmomatic software v0.36 (Bolger et al., 2014) was used to remove sequencing adapters from the reads and low-quality sequence regions with average quality score (Q value) below 15. This step removed reads of <36 bp and kept only paired reads. Each sample was mapped to a combined AR37 endophyte (unpublished) and a *Hordeum*-aligned ryegrass genomic sequence fasta file (Byrne et al., 2015) using STAR v2.5.3a (Dobin et al., 2013). The latter was an in-house version of the reference genome of Byrne et al. (2015), aligned to a *Hordeum vulgare* genome to form pseudochromosomes (Faville et al., 2018). Counts of uniquely mapped read pairs that overlapped known genes (not including ribosomal RNA-encoding genes) were obtained and subjected to differential gene expression analysis between HT and LT treatments. Differentially expressed genes (DEGs) were calculated from counts using R v3.6.1 and edgeR package v3.28 (Robinson et al., 2010) with relative log expression normalization and exact test with Hochberg and Benjamini false discovery rate (FDR) multiple testing correction (Verhoeven et al., 2005). DEGs were defined as genes with values of FDR < 0.05 and fold-change (FC) expression ≥ 1.5 for either the HT>LT or LT>HT treatment comparisons.

### Gene annotation and gene ontology enrichment analyses

Predicted transcript and protein sequences of all genes were annotated using a comprehensive bioinformatics pipeline. Transcripts were queried against the NCBI nr nucleotide database using BLASTN with an e-value cut-off of 1e-20, while protein sequences were queried against the NCBI nr protein and KEGG (Kanehisa and Goto, 2000) databases using BLASTP with an e-value cut-off of 1e-20. InterProScan (Quevillon et al., 2005) was used to identify protein domains and Mercator v3.6 (Lohse et al., 2014) was additionally used (Blast_cutoff: 80) to classify protein sequences, with homology to the available reference plant proteins (TAIR, SwissProt/UniProt, TIGR5 proteins), into MapMan functional plant categories.

An additional analysis of the translated protein sequences using BLASTP against the NCBI nr protein database was performed with a lower e-value cut-off of 1e-10. The output of this search was analyzed in the Blast2GO suite (OmicsBox 1.3.11) to retrieve the gene ontology (GO) annotations of the genes with respect to their putative roles in molecular functions, biological processes and cellular components (Conesa and Götz, 2008). Blast2GO (Conesa et al., 2005) and InterProScan were used to retrieve GO terms for each gene. The GO terms associated with DEGs were analyzed using agriGO v2 (Tian et al., 2017) to identify enriched GO terms by singular enrichment analysis (SEA) compared with a reference gene set based on all of the perennial ryegrass genes annotated in this study. Additionally, at least 10 sets of 200 randomly chosen gene models were subject to GO analysis as above to ensure that there was no bias in SEA results. The statistical method used was Fisher’s test with Hochberg multiple correction adjustment (Benjamini and Yekutieli, 2001), and an adjusted p-value (FDR) of <0.05 for a category was considered significantly enriched. REVIGO (Supek et al., 2011) was used to summarize GO terms from the SEA.

### In silico analysis of putative RLK and R genes

DEGs annotated as encoding receptor-like kinase (RLK) and resistance (R) proteins in the ryegrass reference genome (Byrne et al., 2015) were further manually analyzed using Integrative Genomics Viewer (IGV) (Thorvaldsdóttir et al., 2013) and Geneious Prime v2019.1.1 (Kearse et al., 2012) software. Using IGV, the mapped transcriptome reads were viewed together with gene models relative to their positions on the ryegrass genome scaffolds, and the corresponding cDNA sequences were identified by comparing the reference genome sequence with cDNA sequences in Geneious. The translated protein sequences from Geneious were used for protein domain identification using HMMER (Finn et al., 2011) and InterProScan (Quevillon et al., 2005).

### Genome-wide association study of GBS SNP data

SNP data from the Population IV plants, generated using a GBS approach (Faville et al., 2018; Gagic et al., 2018), were subject to a genome-wide association study (GWAS) to identify associations between genetic regions of the perennial ryegrass genome and the endophyte transmission phenotype. GWAS analysis was implemented using existing SNP data and endophyte transmission rates from the 93 plants of Population IV (Faville et al., 2018; Gagic et al., 2018). Missing values were mean imputed using the “A.mat” function in the rrBLUP R package (Endelman, 2011) and resulted in 1,023,010 SNPs. The GWAS function in rrBLUP was used to fit a mixed linear model, with a previously determined marker derived relationship matrix (K) included in the model to control relatedness within the population (Faville et al., 2018). Since all the plants used in the analysis were from a single population, principal components (Q) were not included as fixed effects in the model to control for population structure, making it a K only model. Population parameters previously determined (P3D) were set to true, as it is a more efficient computational model with similar statistical power compared to the standard mixed linear model (Zhang et al., 2010). GWAS analysis outputs -log_10_p values and were converted to p-values for multiple testing based on Bonferroni correction at a = 0.05 (0.05/1023010 = 4.89e-08). Approximately 1,000 SNPs had p < 0.001 and were considered the most informative markers associated with transmission, and thus were selected for further analysis.

### Functional annotation of genes associated with SNP markers

To directly compare the most highly ranked GWAS SNPs with expressed genes, GMAP v2017.05.08 (Wu et al., 2016) with default settings was used to map the expressed genes to the same reference ryegrass genome assembly used to identify GWAS SNPs, the *Hordeum*-aligned pseudochromosomes (Faville et al., 2018). For SNPs located within, or up to 3 kb from a gene model, GO terms associated with the gene models were obtained. These gene models were also compared to DEGs from the IP and OV tissues to identify SNPs that are linked to DEGs (SNP-DEGs). To test for functional enrichment of GO terms associated with SNPs, the GO terms were compared to the above-mentioned custom reference list using the AgriGO v2 SEA tool as described above using the Fisher’s exact test with Hochberg FDR multiple correction adjustment. Significantly enriched GO terms were referred to as SNP-GOs. Three sets of 1,000 randomly chosen SNPs were analyzed for adjacent gene models and GO terms, and subject to enrichment as above to ensure that there was no bias in enrichment results. The annotations of gene models within significantly enriched GO categories were examined.

### Visualization of QTLs, DEGs and SNPs on the ryegrass linkage map

A phenome map was constructed combining SNP and DEG genetic markers found in the current study, together with quantitative trait loci (QTL) associated with endophyte growth and alkaloid content (Faville et al., 2015), and disease resistance (Dracatos et al., 2008; Pauly et al., 2012; Pfender and Slabaugh, 2013) using the ryegrass GenomeZipper generated by Pfeifer et al. (2013) and Byrne et al. (2015) as a framework. Ryegrass GenomeZipper is a draft putative order of genetic markers and genomic scaffolds of seven linkage groups of *L. perenne*. BLASTN v2.6.0+ (e-value threshold 1e-50) was used to identify the genomic scaffolds containing QTLs. SNP-DEGs, SNP-GOs, and DEGs were located on the linkage maps, plotted on linkage groups, and visualized using PhenoGram plot (http://visualization.ritchielab.org/phenograms/plot).

### Quantification of endophyte biomass and polyphenols in reproductive tissues

To compare the endophyte biomass of the reproductive tissues of HT and LT plant genotypes, in December 2019, spikelets were harvested from newly-emerged inflorescences (spikes) and four spikes per plant were tested. All spikelets of each spike were pooled into one sample, frozen at –20°C, freeze-dried and then subjected to genomic DNA extraction and qPCR analysis of the *E. festucae* non-ribosomal peptide synthetase (NRPS1) gene as previously described by Gagic et al. (2018). A subset of the inflorescence samples was sent to the Laboratory of Organic Chemistry and Chemical Biology in the University of Turku (Turku, Finland) for the analysis of polyphenols. Derivatives of quinic acid, kaempferol, quercetin and myricetin were measured using the method as described by Engstrom et al. (2015).

## Results

### Confirmation of perennial ryegrass AR37 transmission phenotypes

HT and LT perennial ryegrass genotypes were selected for transcriptome analyses based on the endophyte transmission data acquired in seed harvested from the 2013/14 and 2014/15 production seasons (Gagic et al., 2018). Moreover, the stability of the transmission phenotypes of these plant genotypes was confirmed in the seed production season of 2019/20, where HT genotypes showed consistently higher infection frequencies (92 – 100%) than the LT genotypes (37 – 56%) (**Figure 2A**). Two-way ANOVA showed that the seed transmission efficiency was significantly affected by the genotype (p < 0.05) but not between the 2013/14, 2014/15 and 2019/20 seed production seasons (p > 0.05). Examples of the IP and OV tissues that were selected for transcriptome analysis are shown in **Figure 2B**.

### Transcriptome analysis to identify DEGs

A summary of the RNA-Seq of the IP and OV samples from the HT and LT ryegrass genotypes is shown in **Table 1**, with full statistics shown in **Supplementary Table S1**. On average 87.58 million raw read repair counts were obtained per sample, which yielded an average of 87.4 million of quality trimmed read pair counts. The proportion of the trimmed reads that mapped uniquely to the AR37 genome differed between the IP and OV tissues, being higher in the IP (0.35%) than OV samples (0.077%), with a relatively high proportion of these reads mapping to AR37 genes (94 and 84% respectively). In contrast, ~99% of reads mapped uniquely to the ryegrass genome, with 72 and 86% of these reads to ryegrass genes in the IP and OV tissues, respectively. Differential gene expression analyses of HT and LT genotypes from either tissue did not reveal DEGs of endophyte origin, and therefore endophyte DEGs were not explored further in this study.

**Table 1:** Percentage reads mapping to the *Epichloë* sp. LpTG-3 strain AR37 and to the perennial ryegrass genome.

Tissue type	Treatment	Trimmed read pair count (millions)	Percentage mapping to AR37 genome	Percentage mapping to perennial ryegrass genome
IP	HT	95 – 104	0.26 – 0.48	65 – 73
	LT	78 – 86	0.11 – 0.61	65 – 86
OV	HT	84 – 92	0.052 – 0.15	84 – 89
	LT	74 – 86	0.024 – 0.067	72 – 96

IP, inflorescence primordium; OV, ovary; HT, high-transmission; LT, low-transmission.

**Figure 3:**
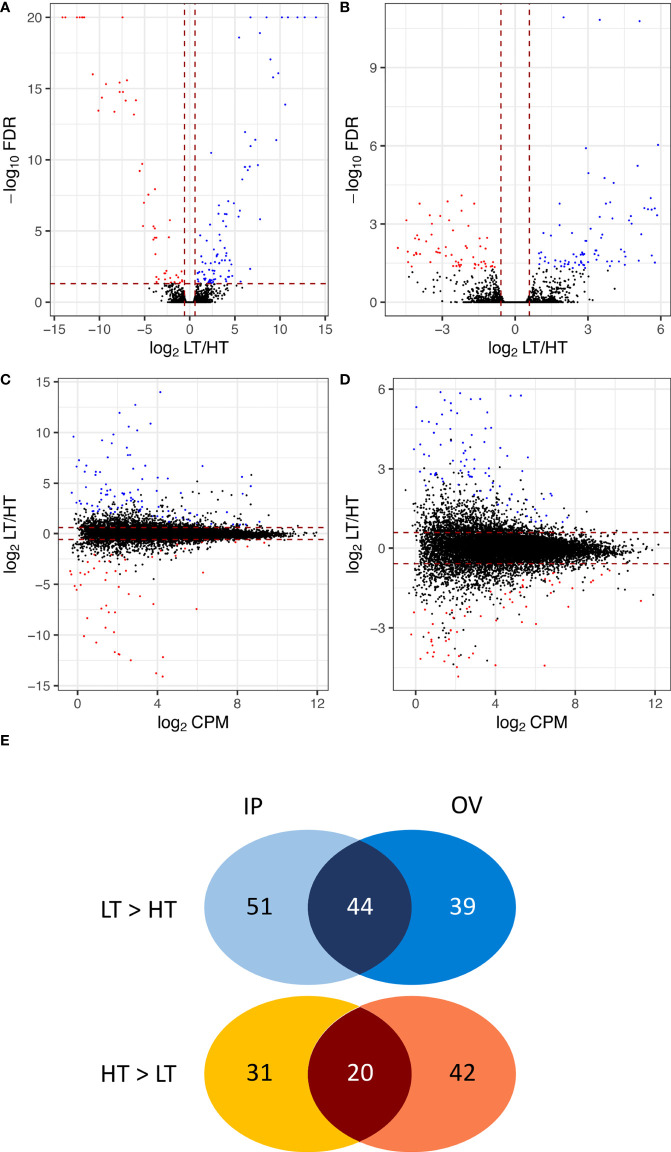
Volcano plots and MA plots showing relative gene expression differences between low-transmission (LT) and high-transmission (HT) genotypes in the **(A, C)** inflorescence primordium (IP), and **(B, D)** ovary (OV) tissues. Venn diagram **(E)** comparing DEGs in the IP and OV tissues, separated into LT>HT and HT>LT expression in each tissue. DEGs are colored blue to indicate those that are upregulated in the LT genotypes and red to represent those that are upregulated in the HT genotypes. DEGs for which -log_10_FDR ≥ 20 were indicated as 20. MA plots comparing the relative gene expression differences between LT and HT genotypes to the average expression (log_2_CPMs) in the **(C)** IP, and **(D)** OV tissues. The DEGs are colored according to the parameters in **(A, B)**.

A total of 25,261 unique plant genes were identified from mapping the trimmed RNA-Seq reads to the *Hordeum*-aligned perennial ryegrass reference genome (Byrne et al., 2015). Overall, 227 DEGs (FDR < 0.05 and FC ≥ 1.5) were identified (**Supplementary Table S2**), where **Figure 3** summarizes the FDR, FC, and relative abundance of genes within the dataset. In the IP samples, 146 DEGs (~ 0.58% of the total genes) were identified between the HT and LT genotypes, with 95 LT>HT and 51 HT>LT DEGs, respectively. In the OV tissues, 145 DEGs (~ 0.57% of the total genes) were identified between the HT and LT genotypes, with 83 LT>HT and 62 HT>LT DEGs, respectively. There were 64 DEGs in common between the IP and OV datasets, with 44 LT>HT and 20 HT>LT, and no DEGs were observed that were LT>HT in one tissue type while HT>LT in the other (**Figure 3**).

### Gene ontology analysis of the HT and LT DEGs

Analysis of the DEGs by AgriGO revealed that 95 and 96 (65.1% and 66.2% of the total) DEGs in the IP and OV samples, respectively, had significant hits to proteins with GO terms, as determined using BLAST2GO (2019). Significantly enriched GO terms in each tissue by SEA, summarized by REVIGO are shown in **Table 2**. The most highly enriched GO term identified was the molecular function, *ADP binding* (GO:0043531), which was found in both the IP and OV datasets, with 8 and 6 LT>HT DEGs, respectively. Several other enriched GO terms identified in the IP tissues were also from genes upregulated in the LT genotypes and were involved in biological processes associated with *response to heat* (GO:0009408), *cellular lipid metabolic process* (GO:0044255) or *polysaccharide metabolic process* (GO:0005976) (**Table 2**). In the OV tissues, many biological processes were upregulated in the HT genotypes, including *response to cold* (GO:0009409), and *monosaccharide metabolic process* (GO:0005996) (**Table 2**). Furthermore, *plant-type cell wall* (GO:0009505) and *intrinsic component of plasma membrane* (GO:0031226) were the highly enriched subcategories under the cellular component category in the OV tissues (**Table 2**).

**Table 2:** Enriched GO terms (p ≤ 0.05) of DEGs in the inflorescence primordium (IP) and ovary (OV) tissues.

Tissue type	Group	Term ID	GO term description	DEGs	
				LT>HT	HT>LT	log_10_P
IP	MF	GO:0043531	ADP binding	8	1	-3.52	
		GO:0016853	isomerase activity	3	2	-1.85	
		GO:0035251	UDP-glycosyltransferase activity	3	0	-1.59	
		GO:0019899	enzyme binding	2	2	-1.37	
	BP	GO:0009408	response to heat	3	1	-2.62	
		GO:0044255	cellular lipid metabolic process	3	3	-1.32	
		GO:0005976	polysaccharide metabolic process	4	1	-1.62	
		- GO:0009250	- glucan biosynthetic process	3	0	-1.75	
OV	MF	GO:0043531	ADP binding	6	0	-2.17	
		GO:0015926	glucosidase activity	2	1	-1.92	
	BP	GO:0043531	protein-containing complex	0	3	-1.62	
		GO:0009266	response to temperature stimulus	0	4	-1.70	
		- GO:0009409	- response to cold	0	3	-1.80	
		GO:0005996	monosaccharide metabolic process	2	1	-1.37	
	CC	GO:0009505	plant-type cell wall	2	1	-1.51	
		GO:0031226	intrinsic component of plasma membrane	2	3	-1.68	

MF, molecular function; BP, biological process; CC, cellular component.

The terms shown in this Figure may have mother terms not shown if they contain the same genes with the child terms.

### Functional categories of DEGs

**Figure 4:**
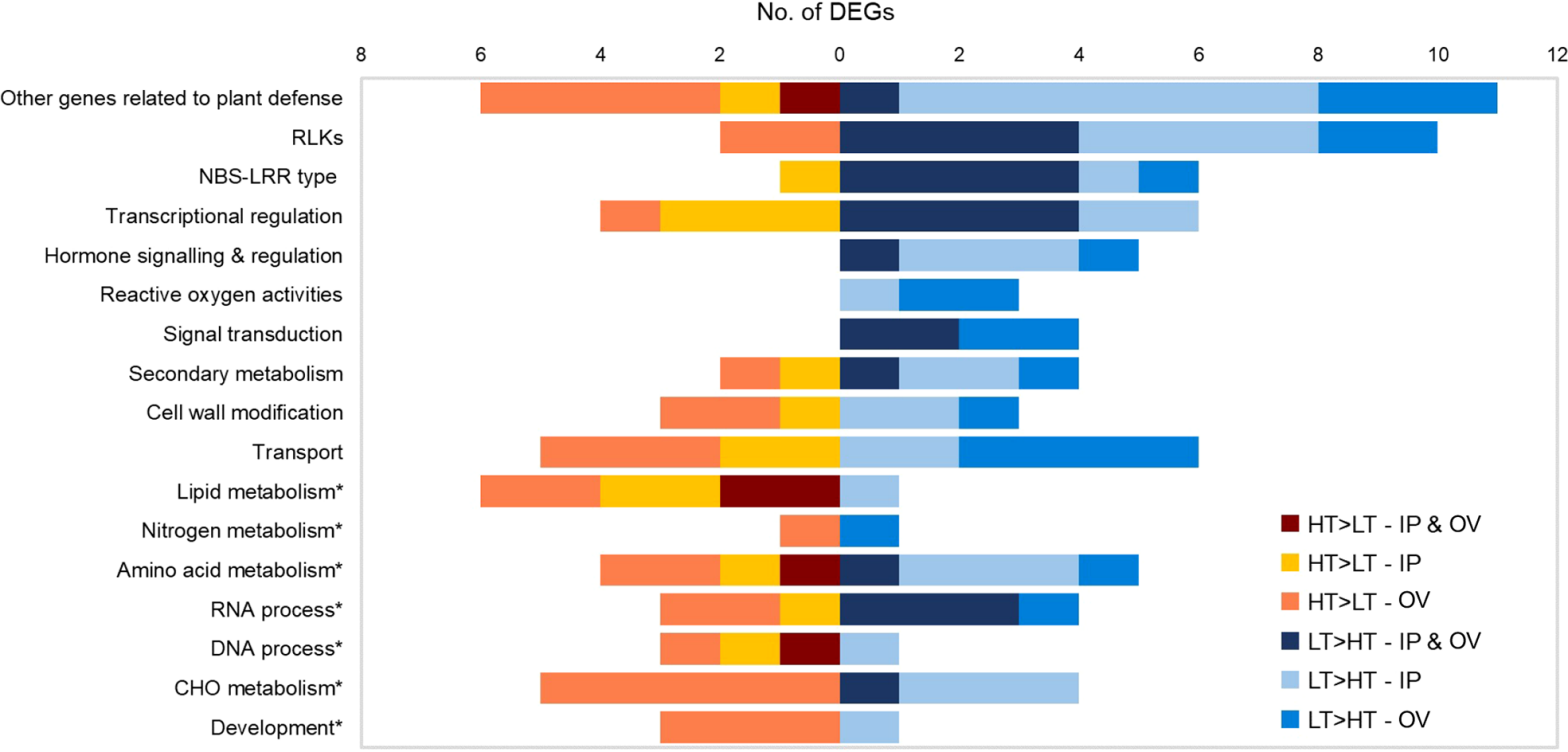
Functional categorization of IP and OV DEGs. Numbers of HT>LT or LT>HT DEGs in each functional category for IP, OV or both tissues (common DEGs). No genes are represented more than once within a given category. IP, inflorescence primordium; OV, ovary; LT, low-transmission and HT, high-transmission. Asterisk denotes primary metabolism categories.

Functional predictions of DEGs were based on manual inspection of BLAST, KEGG, InterProScan and Mercator results (**Supplementary Table S2**). Genes with putative roles in plant defense were frequently identified, thus relevant DEGs (n = 120) were assigned to functional groups including pathogen detection, signal transduction and plant defense responses, based on their general function predictions (Galindo-González and Deyholos, 2016) (**Figure 4**). The most abundant DEGs were predicted to encode RLKs (12 DEGs), extracellular sensors that act in plant defense responses, which were mainly upregulated in the LT plants. DEGs encoding nucleotide binding site-leucine rich repeat (NBS-LRR) proteins, resistance (R) proteins associated with intracellular pathogen detection, were also abundant and were mainly upregulated in the LT plants of both IP and OV tissues. DEGs associated with signaling, transcriptional regulation and reactive oxygen processes were largely upregulated in the LT plants, as well as other genes related to plant defense (e.g. heat shock proteins (Hsp), ubiquitin-protein ligase and proteinase inhibitors). In contrast, DEGs upregulated in HT plants included those involved in lipid metabolism and amino acid metabolism (**Figure 4**).

Given the predominance of DEGs that were assigned as RLK and R genes, we examined the protein domain structures of these to confirm their likely functions as plant immune receptors. The RLK proteins are a diverse superfamily (Dievart et al., 2020). All of the differentially expressed RLK genes observed in this study contained kinase domains, though only some contained identifiable LRR or transmembrane domains (**Supplementary Figure S1**). These putative RLK genes were all affiliated with the *ATP binding* (GO:0005524) *via* Blast2GO analysis. In contrast, all of the putative R genes encoded coiled coil (CC) domains at the N-terminus (except for gene 0025972-0.4), an NB-ARC domain, and LRR domain at the C-terminus, typical of R genes (**Supplementary Figure S1**) (Dracatos et al., 2009). Furthermore, the R genes with NB-ARC domains were all associated with the *ADP binding* (GO:0043531) GO term. Ten out of the 12 putative RLK genes and 6 out of the 7 putative R genes were upregulated in the LT compared to HT tissues, with 4 RLK genes and 4 R genes being upregulated in both tissues (**Figure 4**). Two of the putative RLK genes were downregulated in the OV samples only, and these possessed a single LRR domain and protein kinase domain (**Supplementary Figure S1**). One differentially expressed R gene (0009038-0.0, LT>HT in both IP and OV) was identified as a homolog of *Arabidopsis* at3g07040 (Jung et al., 2020), which encodes an RPM1-induced protein kinase (RPM1) that specifically recognizes the AvrRpm1 type III effector avirulence protein from *Pseudomonas syringae* (Liu et al., 2011). The putative R gene DEG that lacked a CC domain, 0025972-0.4 (upregulated in both IP and OV) had similarity to an R protein RPP13 in *Arabidopsis* (at3g46530) with a putative amino-terminal leucine zipper that confers resistance against the biotrophic phytopathogen, *Peronospora parasitica* (**Supplementary Table S2**) (Bittner-Eddy et al., 2000). The putative RLK DEG (0043343-0.3) was predicted to encode a lectin kinase, and had homology to the *Arabidopsis* gene at4g21390, thought to be involved in microbe recognition and defense signaling (Meier et al., 2010).

### SNP-associated gene model enrichment

GWAS analysis of the SNP data generated by GBS from 93 Population IV parent plants identified 1000 SNPs that were highly associated (p < 0.001) with the AR37 transmission phenotype. These SNPs were examined for their proximity to ryegrass gene models and the DEGs identified by transcriptome analysis of the IP and OV samples, and for enriched GO term categories from the associated gene models. Of the 1000 SNPs, 392 were found to be within 3 kb of a ryegrass gene model, and in total, 214 unique gene models were identified. Approximately one third of these gene models had multiple (2-11) independent SNPs associated with them. Furthermore, one gene (0043153-0.0) was also a DEG in the OV samples only and was upregulated 12.7-fold in the HT as compared to LT group, where it had low expression (**Supplementary Table S2**). This DEG is predicted to encode a mechanosensitive ion channel protein.

SEA of the 174 SNP-associated gene models that had GO term assignments, revealed the enrichment of several GO categories (p < 0.05, **Table 3**), with two highly significant (Hochberg adjusted p < 0.05) and one trending toward enrichment (adj. p = 0.061) cellular process categories: *defence response to fungus* (GO:0050832), *response to fungus* (GO:0009620) and *defence response* (GO:0006952), respectively. Ten SNP-associated genes were assigned to these GO terms (i.e., SNP-GO genes), which included genes with multiple SNPs near the gene (**Supplementary Table S3**). These analyses provide further evidence that host defense plays a key role in influencing the vertical transmission efficiency of AR37.

**Table 3:** Enriched GO terms (p ≤ 0.05) from 174/230 gene models with GO annotation that were associated with GWAS single nucleotide polymorphisms (SNP).

Group	Term ID	GO term description	no. SNP associated gene models	log_10_P
MF	GO:0048037	cofactor binding	11	-2.13
BP	GO:0043207	response to external biotic stimulus	7	-2.11
	- GO:0009620	- response to fungus	6	-3.35
	GO:0006952	defence response	10	-2.85
	– GO:0050832	– defence response to fungus	6	-4.00
	GO:0006511	ubiquitin-dependent protein catabolic process	8	-1.70
	GO:0044248	cellular catabolic process	16	-1.49
CC	GO:0098805	whole membrane	10	-1.52

MF, GO molecular function; BP, biological process; CC, cellular component.

The terms shown in this Figure may have mother terms not shown if they contain the same genes with the child terms.

### Genetic linkage of DEGs, SNP-GO genes associated with plant defense

**Figure 5:**
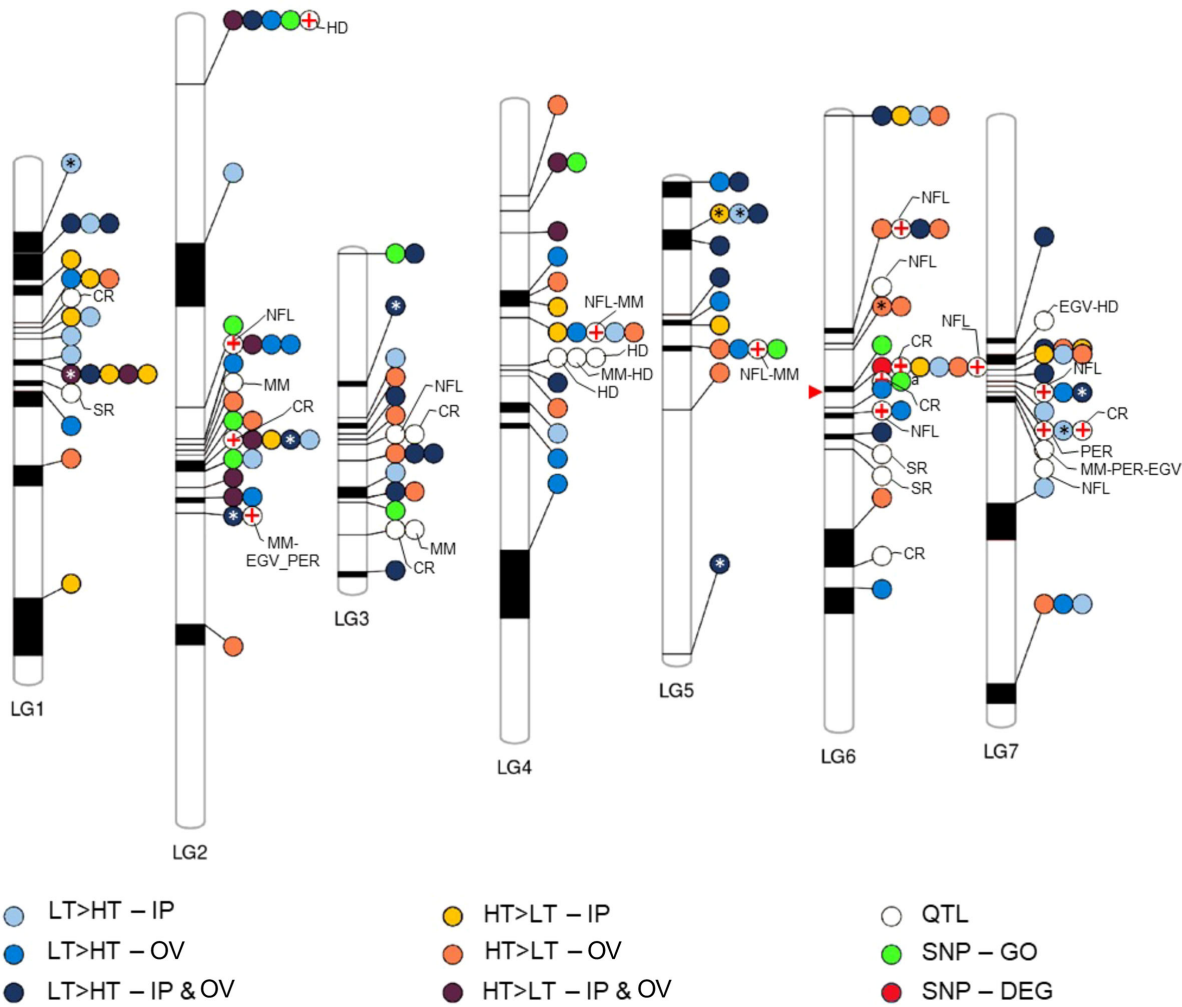
Co-location of genetic features of interest to this study. Phenogram showing the relative position ranges of DEGs, QTLs, and SNPs on the linkage group map assembled by Byrne et al. (2015). Asterisks indicate DEGs that are annotated as disease resistance genes, while red crosses denote QTLs that approximately co-locate on a linkage group with features identified in this study. The red arrow points to a prominent cluster of co-located features including, SNP-DEG, SNP-GO, DEGs and QTLs, wherein the crown rust (CR) QTL and DEG (yellow dot) co-locate on the same genomic scaffold. Sizes of linkage groups (LG1-7) are 97.7, 151.5, 63.3, 119.2, 90, 115.2, and 113.7 centimorgan respectively. EGV, ergovaline; HD, heading date; MM, mycelial mass; NFL, N-formyl loline; PER, peramine; SR, stem rust (*Puccinia graminis*).

We assessed the physical relationships between the plant defense associated DEGs, and SNP-GO genes within the perennial ryegrass genome based on a genetic linkage map against which genome scaffolds had been mapped (Byrne et al., 2015). Furthermore, we determined the positions of the DEGs and SNP-GO genes relative to established QTL markers for disease resistance and endophyte biomass (Faville et al., 2015). This resulted in the linkage map shown in **Figure 5**, which contained 113 DEGs, one SNP-DEG (0043153-0.0), 10 SNP-GO genes and 31 QTLs distributed on 7 linkage groups. A prominent cluster containing a SNP-DEG, a SNP-GO gene, several DEGs and QTLs was located on linkage group 6. A QTL for resistance to crown rust (*Puccinia coronata*) (Dracatos et al., 2008; Pauly et al., 2012) mapped to the same genomic scaffold as a DEG (0047557-0.1) that was upregulated in the IP of HT plants and predicted to encode an M48-type endopeptidase involved in protein degradation. This was the only such occurrence of a DEG or SNP-GO gene that was co-located on the same scaffold. Many scaffolds (ca. 50%) were not mapped to linkage groups (Byrne et al., 2015), and the exact order and distance between scaffolds has not been previously validated in perennial ryegrass. However, several differentially expressed R genes appeared to be co-located on linkage groups with QTL markers, particularly those associated with endophyte alkaloid production (**Figure 5**). From the GWAS variable importance in projection cores of the SNP markers associated with transmission, none stood out as more highly informative than others, which may suggest that multiple genetic loci and functions are associated with the transmission phenotype.

### Relationship between endophyte biomass, polyphenols and seed transmission in reproductive tissues

**Figure 6:**
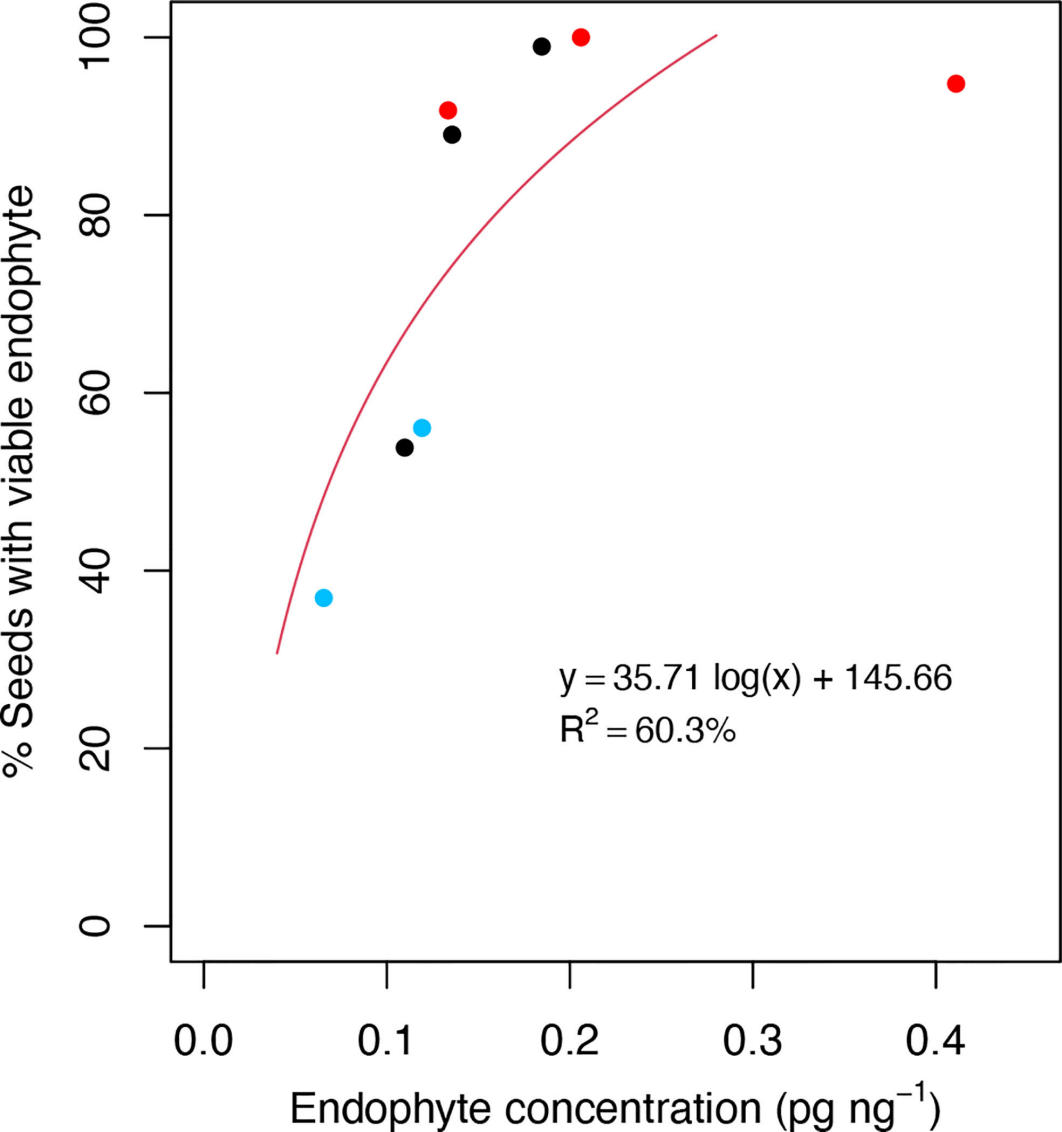
Scatterplots of the % viable endophyte infection rate in seeds relative to endophyte DNA concentration in spikelet tissues. Observations were taken from a range of high-transmission (HT) and low-transmission (LT) genotypes from Population IV that were assessed for % viable endophyte transmission rate in the seed harvest season of 2019/20, and endophyte concentration was determined from spikelet tissues from the same season. Red and blue dots represent HT and LT genotypes used for the transcriptome analysis, respectively. Black dots represent genotypes not used for the transcriptome analysis. Each dot represents the averaged data from triplicate measurements from the same genomic DNA preparation by qPCR.

Given the association of plant defense processes with *Epichloë* transmission, we explored the possibility that LT perennial ryegrass genotypes may suppress endophyte growth in the host reproductive tissues as compared to HT genotypes and result in lower endophyte biomass in these tissues. We quantified the biomass of AR37 within the spikelets of the five genotypes used for transcriptome analyses, as well as three additional genotypes (70, 108, 17) from Population IV to span a wide range of vertical transmission frequencies (37−100%). Additional genotypes could not be assessed due to lack of flowering the season they were examined (2019/20). These genotypes included five genotypes previously used in this study for transcriptome analysis and a further three genotypes from Population IV (HT: 70, 108, LT: 17). Regression analysis showed a positive relationship between fungal biomass and vertical endophyte transmission (**Figure 6**) (y = 35.71 log(x) + 145.66, R^2^ = 60.3%). The model fit for the regression equation assessed through F statistics was significant (p = 0.023).

In the reproductive tissues of ryegrass plants, derivatives of quinic acid, kaempferol and quercetin were detected, but not those of myricetin. the concentrations of kaempferol derivatives, quercetin derivatives, flavonol derivatives and polyphenol derivatives were generally higher on average in the HT than LT group, though no significant difference was observed (p > 0.05 by the Kruskal-Wallis test) (**Supplementary Table S4**). Moreover, correlations between the compound concentrations and either transmission rate or endophyte biomass (indicated by endophyte DNA concentration) were not apparent.

## Discussion

**Figure 7:**
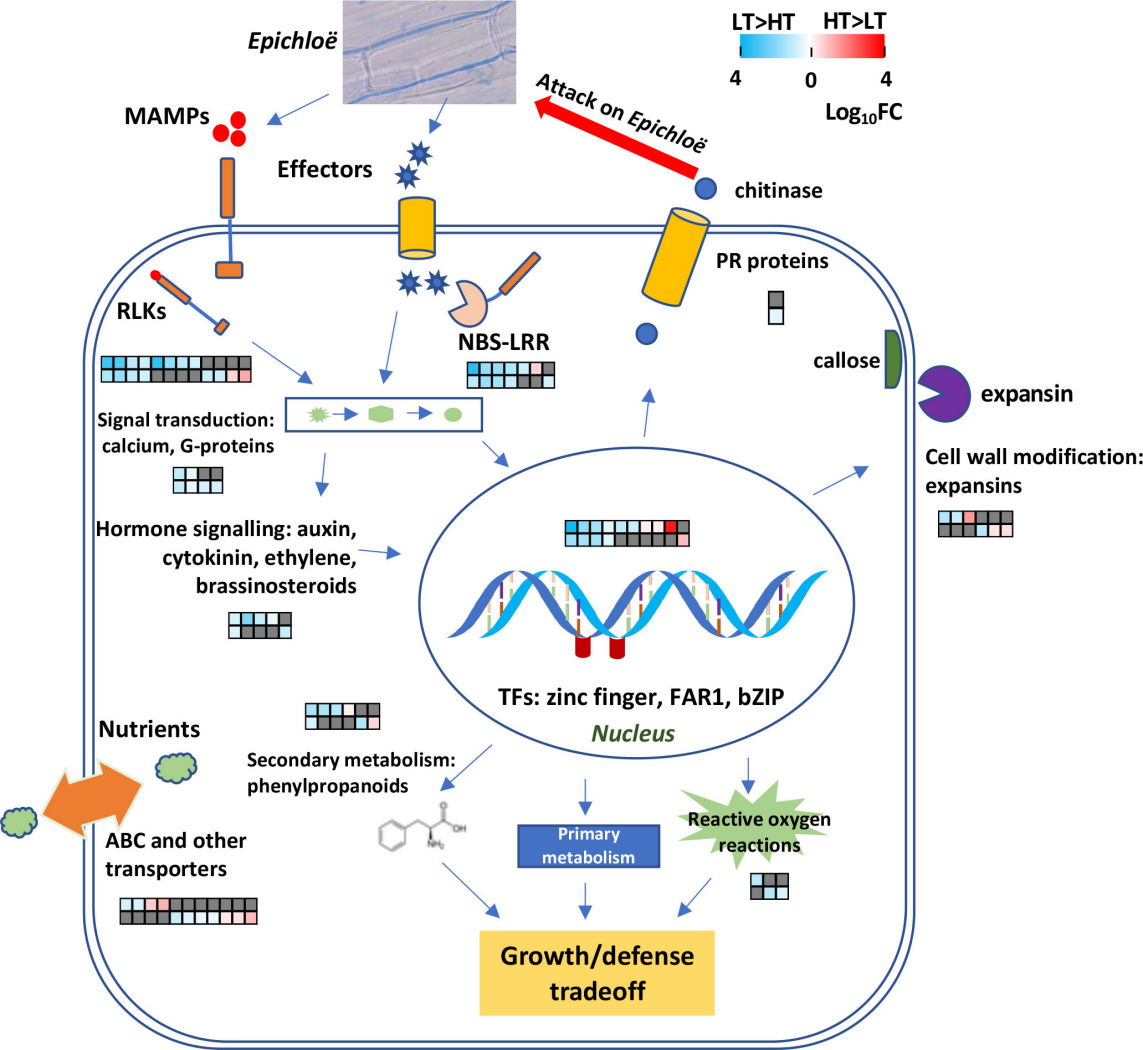
A model illustrating host defense responses to *Epichloë* spp. infection. Differentially expressed genes (DEGs) between high-transmission (HT) and low-transmission (LT) genotypes are depicted in heatmaps. The data are displayed as heatmaps of log_10_FC values, with LT>HT DEGs highlighted in blue and HT>LT DEGs in red, and non-DEGs in dark grey. Each column corresponds to the same gene in the IP and OV tissues. The heatmap’s upper row depicts DEGs in the IP while the lower row depicts DEGs in the OV tissues.

Our transcriptomic analyses of the IP and OV tissues from HT and LT perennial ryegrass genotypes revealed the differential expression of 70 genes predicted to be associated with plant disease resistance mechanisms, including processes involved in pathogen detection, signaling and defense responses (see **Figure 7** for schematic model). Despite the IP and OV tissues being sampled at different growth stages, many of these genes (>70%) were upregulated in both tissues of LT hosts, highlighting relatively consistent responses in plant defense in relation to endophyte transmission frequencies within these genotypes. Most notably, 19 RLK and R genes, likely involved in host detection of endophyte, were differentially expressed. This suggests that the host genotypes associated with HT and LT behave differently when perceiving and interacting with AR37, with LT genotypes being more resistant to AR37 infection. Furthermore, in an independent analysis of 93 Population IV perennial ryegrass genotypes, we found SNPs that were associated with genes with enriched GO terms that describe defense responses to fungi. Moreover, we detected one gene model (0043153-0.0) in common between the GWAS and transcriptome data, that was upregulated in the OV samples of the HT plants. This gene is predicted to encode a putative mechanosensitive ion channel protein, which is broadly involved in plant signaling in response to diverse stimuli such as osmotic pressure and pathogenic invasion (Basu and Haswell, 2017) and may contribute to the greater endophyte biomass in the HT plant reproductive tissues. Together, these analyses provide strong support for an integral role for plant defense genes in the vertical transmission of *Epichloë*. Consistent with this view, endophyte biomass was significantly lower in the reproductive tissues of LT genotypes compared to HT genotypes, implying the growth of AR37 was inhibited in LT genotypes.

Within the LT genotypes, many genes that have been traditionally associated with pathogen detection had large degrees of upregulation compared to the HT genotypes (**Figure 7**). However, it is unclear whether the HT genotypes are likely to be less responsive to pathogens compared to LT genotypes. For many plant species, the first inducible line of defense against phytopathogens is activated by pattern recognition receptors that recognize microbe-associated molecular patterns (MAMPs) (Thoms et al., 2021) and include chitin and ergosterol, common components of fungal hyphae. Chitin promotes host PAMP-triggered immunity (PTI) that activates plant chitinase genes and the release of chitin oligosaccharides (Kombrink et al., 2011).

A recent study indicated that the cell walls of *Epichloë festucae* are remodeled from chitin to chitosan during the early infection stage of grass seedlings. This is postulated to help evade PTI and maintain mutualistic symbiosis (Noorifar et al., 2021). Our current study identified ten RLK genes upregulated in the LT plants (**Supplementary Figure S1**) and these may have roles in binding to MAMPs from *Epichloë* spp. Additionally, a putative somatic-embryogenesis receptor-like kinase 1 gene (SERK1) was co-located with a population level SNP marker identified through GWAS analysis. In terms of mutualistic associations between plants and rhizobacteria or mycorrhizal fungi, several receptor kinases (SymRK), such as LysM receptor-like kinases and Lyk3, with key roles in compatibility have been identified (Gherbi et al., 2008; Haney et al., 2011). It is possible that the two HT>LT RLKs identified from this study play a role in maintaining a mutualistic symbiosis with *Epichloë*, but further characterization is required. Also, fungi produce effectors to promote colonization of their hosts whilst hosts use R proteins to identify the effectors and induce effector-triggered immunity. Only a few effectors with significant roles in mutualistic interactions have been described to date, and include those from *Epichloë* spp. (Hassing et al., 2019; Wang et al., 2021). We identified seven differentially-expressed R genes. However, it is unknown to which effectors their products bind to, or indeed, if they do bind effectors in this interaction.

Upon pathogen detection, host plants commonly regulate the expression of pathogenesis-related (PR) genes through a range of signaling mechanisms (Sels et al., 2008). Only one serine/threonine protein kinase, involved in the common/widespread mitogen-activated protein kinase (MAPK) signal transduction cascade (Morris, 2001), was differentially regulated, being upregulated in LT hosts in both IP and OV samples. However, two DEGs associated with G-protein signaling were upregulated in the LT hosts in both tissues, one of which was 9-fold overexpressed. Plant G-proteins are associated with atypical receptors and receptor-like kinases and are thought to act *via* phosphorylation and de-phosphorylation (Trusov and Botella, 2016). One DEG involved in calcium signaling was 5-fold overexpressed in LT>HT genotypes within the OV samples. Calcium-dependent secondary messenger signaling is a major pathway for signal transduction perceived by cell surface receptors such as G-protein coupled receptors and histidine kinases (Eaton et al., 2012). The two most important hormonal signaling pathways associated with induced plant defenses are the jasmonic acid and salicylic acid pathways. However, DEGs related to either of these pathways were not detected, despite observing a LT>HT IP DEG associated with a gene encoding multiprotein-bridging factor 1a (MBF1a). Overexpression of MBF1a enhances fungal infection *via* mediation of the ethylene/jasmonic acid-response signal transduction pathway in *Arabidopsis* (Kim et al., 2007). Intriguingly, three DEGs that were all strongly upregulated in LT (up to 68-fold) are described as FAR1-related sequence 5-like proteins, which are putative transcriptional activators involved in regulating light control of growth and development in *Arabidopsis*. Their roles in *Epichloë*-infected reproductive tissues are currently unknown. The downregulation of WRKY transcription factor genes in endophyte-infected compared to endophyte-free plants, has been observed in endophyte-infected compared to endophyte-free hosts, both naturally and artificially, and is involved in disease and abiotic stress responses (Dupont et al., 2015; Dinkins et al., 2017), but these were not found to be differentially regulated in our study.

Among classical defense responses, genes involved in ROS metabolism, cell wall modifications and secondary metabolism were differentially expressed, many of which were upregulated in the LT genotypes. The upregulation of these mRNA levels is consistent with the lower endophyte biomass detected in the reproductive tissues of perennial ryegrass. Modifications to plant cell walls are often associated with plant defense responses (Malinovsky et al., 2014), and we observed two callose synthase genes upregulated in the IP of the LT plants, whereas an expansin gene was upregulated in the OV samples of the HT plants. Callose deposited between the plasma membrane and cell wall has been hypothesized to provide a physical barrier during pathogen infection (Chen and Kim, 2009). In contrast, expansins non-enzymatically loosen plant cell walls, thus increasing cell plasticity (Cosgrove, 2000), which has been associated with plants infected by beneficial microorganisms, compared to non-infected plants (Giordano and Hirsch, 2004; Dermatsev et al., 2010).

Strikingly, several genes encoding ubiquitin-protein ligase and proteasome-associated genes were upregulated in both IP and OV samples and these genes were also identified by GWAS analysis. The ubiquitin-proteasome system post-translationally modifies proteins by tagging them with ubiquitin, resulting in their degradation by the proteasome. This system is implicated in the regulation of the plant immune system *via* salicylic acid (Adams and Spoel, 2018). Highly conserved molecular (co-)chaperone provide stabilizing interactions during client protein maturation and corrective refolding, and are involved in activating signaling proteins (Didelot et al., 2006). This study identified three DEGs encoding Hsps (Hsp20, Hsp90, Hsp101) and one co-chaperone, an Hsp70/Hsp90 organizing protein (Hop/Sti1), all LT>HT (>15-fold) within the IP samples. Tomato Hsp20 is involved in resistance to *Fusarium oxysporum via* stabilizing interactions with the host resistance protein, I-2 (van Ooijen et al., 2010). Hsp90 proteins are involved in plant disease resistance signaling in response to stress (Kanzaki et al., 2003). In plants, immune sensing NLR proteins are client proteins of Hsp90, mediated *via* co-chaperones SGT1 and RAR1 (Kadota and Shirasu, 2012), and including R proteins RPM1, N and Rx (Hubert et al., 2003; Liu et al., 2004; Jelenska et al., 2010). Concomitantly, expression of the ryegrass putative RPM1 was upregulated in LT IP suggesting chaperone-R protein interactions may contribute towards stabilization of certain R proteins, and to the LT phenotype.

Secondary metabolites play important roles in plant defense reactions against phytopathogens with many of these compounds derived from the phenylpropanoid and flavonoid pathway. We identified five DEGs involved in secondary metabolite production, including three DEGs encoding E-class cytochrome P450 oxygenases. Notably, LT genotype samples exhibited increased gene expression of chalcone isomerase (CHI), anthranilate N-benzoyltransferase, and two of the P450 encoding genes involved in the biosynthesis of flavonoids, particularly phytoalexins: CHI is a key early biosynthetic enzyme of flavonoid/isoflavonoid biosynthesis (Ralston et al., 2005) and anthranilate N-benzoyltransferase is a conserved protein catalyzing the first committed step in biosynthesis of phytoalexin methoxydianthramide B (Reinhard and Matern, 1989). The two P450 proteins belong to CYP71 flavonoid monooxgenases which contribute towards sesquiterpene biosynthesis, with highest BLASTP homologies to Triticeae P450 enzymes whose counterparts in characterized species have phytoalexin products (Takahashi et al., 2007; Poloni and Schirawski, 2014; Liu et al., 2020). Previous studies noted that numerous DEGs involved in lignin and anthocyanin branches of the phenylpropanoid pathway were induced by *E. festucae*-infection, with corresponding increases in the concentrations of anthocyanins and flavonoids (Dupont et al., 2015). Although, differences between the HT and LT genotypes with respect to concentrations of quinic acid, kaempferol, quercetin, flavonol and polyphenol derivatives were not apparent in this study (**Supplementary Table S4**), further work is needed to determine whether a phytoalexin that is unknown to us is produced.

A limitation of the current study is that the ryegrass reference genome used for the transcriptome and SNP analyses is 40% complete. Despite this, 87% of the transcriptome reads mapped to the reference genome, suggesting that its degree of completion did not affect the results unduly. Nonetheless, it is possible that other DEGs related to plant defense, or other functions, may have been missed. In addition, we were not able to examine the AR37 transcriptome due to a combination of low hyphal biomass and high variability in sequence reads in our samples. A greater depth of sequencing will likely enable greater insights into the contribution of the fungal symbiont to the seed transmission outcomes in these host genotypes. We also plan further research to examine the relationship between endophyte effector gene expression in host genotypes with different endophyte transmission frequencies.

The efficiency of AR37 transmission to seed by its ryegrass host is strongly influenced by the perennial ryegrass genotype. This study revealed that the host responses to the perception of endophyte, along with downstream activation of plant defense-associated systems within host reproductive tissues, are likely contributors to decreased endophyte biomass and vertical transmission frequencies. Additionally, gene loci associated with endophyte vertical transmission efficiency have been identified and are enriched for functions associated with plant response to fungi. While several aspects of these findings require further investigation, this study has produced a clear overview of coordination between multiple systems, as shown in **Figure 7**. It seems most likely that endophyte seed-transmission efficiency is influenced by nuanced host-genotype defense responses, which in turn, control the degree of endophyte colonization in host reproductive tissues. Genes or SNPs identified in this study could be developed as markers for use, individually or in combination, in marker-assisted selection or genomic selection schemes, for selecting and breeding grass plants with better *Epichloë* seed transmission.

## Data availability statement

The datasets presented in this study can be found in online repositories BioProject PRJNA732816. The names of the repository/repositories NCBI and accession number(s) can be found in the article/**Supplementary Material**.

## Author contributions

CRV, MG, RDJ, LJJ, CM, WZ and NTF designed the research. WZ, NTF, MG performed the experimental work; NTF, PHM, WZ, SKA, CDM, CRV analyzed the data. CRV, WZ, CDM, NTF, MJF interpreted the data. CDM, WZ, NTF, PHM, SKA, CM, SDC and CRV wrote the manuscript. All authors contributed to the article and approved the submitted version.

## Funding

This research was supported by the New Zealand Ministry of Business, Innovation and Employment Strategic Science Investment Fund and Core funding to AgResearch, as well as the Massey University Research Fund.

## Acknowledgments

We thank Emma Applegate for technical support with the qPCR analysis; Siva Ganesh and Ruy Jauregui for early discussion of the GBS data; and David Hume for critically reviewing this manuscript. We thank School of Agriculture and Environment, Massey University and China Scholarship Council for support of WZ.

## Conflict of interest

WZ, NTF, CDM, PHM, MG, SKA, SDC, RDJ, LJJ, MJF and CRV are/were employed by AgResearch Ltd., New Zealand.

The remaining authors declare that the research was conducted in the absence of any commercial or financial relationships that could be construed as a potential conflict of interest.

## Publisher’s note

All claims expressed in this article are solely those of the authors and do not necessarily represent those of their affiliated organizations, or those of the publisher, the editors and the reviewers. Any product that may be evaluated in this article, or claim that may be made by its manufacturer, is not guaranteed or endorsed by the publisher.

## References

[B1] Adams E. H. G. Spoel S. H. (2018). The ubiquitin-proteasome system as a transcriptional regulator of plant immunity. J. Exp. Bot. 69, 4529–4537. doi: 10.1093/jxb/ery216 29873762

[B2] Bacon C. W. Porter J. K. Robbins J. D. Luttrell E. S. (1977). *Epichloë typhina* from toxic tall fescue grasses. Appl. Environ. Microbiol. 34, 576–581. doi: 10.1128/aem.34.5.576-581.1977 931377 PMC242703

[B3] Basu D. Haswell E. S. (2017). Plant mechanosensitive ion channels: an ocean of possibilities. Curr. Opin. Plant Biol. 40, 43–48. doi: 10.1016/j.pbi.2017.07.002 28750206 PMC5714682

[B4] Benjamini Y. Yekutieli D. (2001). The control of the false discovery rate in multiple testing under dependency. Ann. Stat. 29, 1165–1188. doi: 10.1214/aos/1013699998

[B5] Bittner-Eddy P. D. Crute I. R. Holub E. B. Beynon J. L. (2000). RPP13 is a simple locus in *Arabidopsis thaliana* for alleles that specify downy mildew resistance to different avirulence determinants in *Peronospora parasitica* . Plant J. 21, 177–188. doi: 10.1046/j.1365-313x.2000.00664.x 10743658

[B6] Bolger A. M. Lohse M. Usadel B. (2014). Trimmomatic: a flexible trimmer for illumina sequence data. Bioinformatics 30, 2114–2120. doi: 10.1093/bioinformatics/btu170 24695404 PMC4103590

[B7] Byrne S. L. Nagy I. Pfeifer M. Armstead I. Swain S. Studer B. . (2015). A synteny-based draft genome sequence of the forage grass *Lolium perenne* . Plant J. 84, 816–826. doi: 10.1111/tpj.13037 26408275

[B8] Caradus J. R. Card S. D. Hewitt K. G. Hume D. E. Johnson L. J. (2021). Asexual *Epichloë* fungi–obligate mutualists. Encyclopedia 1, 1084–1100. doi: 10.3390/encyclopedia1040083

[B9] Chen X.-Y. Kim J.-Y. (2009). Callose synthesis in higher plants. Plant Signal. Behav. 4, 489–492. doi: 10.4161/psb.4.6.8359 19816126 PMC2688293

[B10] Christensen M. J. Bennett R. J. Ansari H. A. Koga H. Johnson R. D. Bryan G. T. . (2008). *Epichloë* endophytes grow by intercalary hyphal extension in elongating grass leaves. Fungal Genet. Biol. 45, 84–93. doi: 10.1016/j.fgb.2007.07.013 17919950

[B11] Christensen M. J. Voisey C. R. (2009). “Tall fescue-endophyte symbiosis,” in Tall fescue for the twenty-first century. Eds. Fribourg H. A. Hannaway D. B. West C. P. (Madison WI, USA: American Society of Agronomy, Crop Science Society of America, Soil Science Society of America), 251–272. doi: 10.2134/agronmonogr53.c14

[B12] Conesa A. Götz S. (2008). Blast2GO: A comprehensive suite for functional analysis in plant genomics. J. Biol. Chem. 2008, 679832. doi: 10.1155/2008/619832 PMC2375974 18483572

[B13] Conesa A. Götz S. García-Gómez J. M. Terol J. Talón M. Robles M. (2005). Blast2GO: A universal tool for annotation, visualization and analysis in functional genomics research. Bioinformatics 21, 3674–3676. doi: 10.1093/bioinformatics/bti610 16081474

[B14] Cosgrove D. J. (2000). Loosening of plant cell walls by expansins. Nature 407, 321–326. doi: 10.1038/35030000 11014181

[B15] Dermatsev V. Weingarten-Baror C. Resnick N. Gadkar V. Wininger S. Kolotilin I. . (2010). Microarray analysis and functional tests suggest the involvement of expansins in the early stages of symbiosis of the arbuscular mycorrhizal fungus *Glomus intraradices* on tomato (*Solanum lycopersicum*). Mol. Plant Pathol. 11, 121–135. doi: 10.1111/j.1364-3703.2009.00581.x 20078781 PMC6640415

[B16] Didelot C. Schmitt E. Brunet M. Maingret L. Parcellier A. Garrido C. (2006). “Heat shock proteins: endogenous modulators of apoptotic cell death,” in Molecular chaperones in health and disease (Heidelberg, Germany: Springer), 171–198. doi: 10.1007/3-540-29717-0_8 16610360

[B17] Dievart A. Gottin C. Périn C. Ranwez V. Chantret N. (2020). Origin and diversity of plant receptor-like kinases. Annu. Rev. Plant Biol. 71, 131–156. doi: 10.1146/annurev-arplant-073019-025927 32186895

[B18] Dinkins R. D. Nagabhyru P. Graham M. A. Boykin D. Schardl C. L. (2017). Transcriptome response of *Lolium arundinaceum* to its fungal endophyte *Epichloë coenophiala* . New Phytol. 213, 324–337. doi: 10.1111/nph.14103 27477008

[B19] Dobin A. Davis C. A. Schlesinger F. Drenkow J. Zaleski C. Jha S. . (2013). STAR: Ultrafast universal RNA-seq aligner. Bioinformatics 29, 15–21. doi: 10.1093/bioinformatics/bts635 23104886 PMC3530905

[B20] Dracatos P. M. Cogan N. O. I. Dobrowolski M. P. Sawbridge T. I. Spangenberg G. C. Smith K. F. . (2008). Discovery and genetic mapping of single nucleotide polymorphisms in candidate genes for pathogen defence response in perennial ryegrass (*Lolium perenne* l.). Theor. Appl. Genet. 117, 203–219. doi: 10.1007/s00122-008-0766-7 18446316

[B21] Dracatos P. M. Cogan N. O. I. Sawbridge T. I. Gendall A. R. Smith K. F. Spangenberg G. C. . (2009). Molecular characterisation and genetic mapping of candidate genes for qualitative disease resistance in perennial ryegrass (*Lolium perenne* l.). BMC Plant Biol. 9, 62. doi: 10.1186/1471-2229-9-62 19450286 PMC2694799

[B22] Dupont P. Y. Eaton C. J. Wargent J. J. Fechtner S. Solomon P. Schmid J. . (2015). Fungal endophyte infection of ryegrass reprograms host metabolism and alters development. New Phytol. 208, 1227–1240. doi: 10.1111/nph.13614 26305687 PMC5049663

[B23] Eaton C. Mitic M. Scott B. (2012). “Signalling in the epichloë festucae: perennial ryegrass mutualistic symbiotic interaction,” in Signaling and communication in plant symbiosis (Heidelberg, Germany: Springer), 143–181. doi: 10.1007/978-3-642-20966-6_7

[B24] Endelman J. B. (2011). Ridge regression and other kernels for genomic selection with r package rrBLUP. Plant Genome 4, 250–255. doi: 10.3835/plantgenome2011.08.0024

[B25] Engstrom M. T. Palijarvi M. Salminen J.-P. (2015). Rapid fingerprint analysis of plant extracts for ellagitannins, gallic acid, and quinic acid derivatives and quercetin-, kaempferol-and myricetin-based flavonol glycosides by UPLC-QqQ-MS/MS. J. Agric. Food Chem. 63, 4068–4079. doi: 10.1021/acs.jafc.5b00595 25853372

[B26] Faville M. J. Briggs L. Cao M. Koulman A. Jahufer M. Z. Koolaard J. . (2015). A QTL analysis of host plant effects on fungal endophyte biomass and alkaloid expression in perennial ryegrass. Mol. Breed. 35, 1–18. doi: 10.1007/s11032-015-0350-1 PMC4506467 26203296

[B27] Faville M. J. Ganesh S. Cao M. Jahufer M. Z. Z. Bilton T. P. Easton H. S. . (2018). Predictive ability of genomic selection models in a multi-population perennial ryegrass training set using genotyping-by-sequencing. Theor. Appl. Genet. 131, 703–720. doi: 10.1007/s00122-017-3030-1 29264625 PMC5814531

[B28] Finn R. D. Clements J. Eddy S. R. (2011). HMMER web server: Interactive sequence similarity searching. Nucleic Acids Res. 39, W29–W37. doi: 10.1093/nar/gkr367 21593126 PMC3125773

[B29] Gagic M. Faville M. J. Zhang W. Forester N. T. Rolston M. P. Johnson R. D. . (2018). Seed transmission of *Epichloë* endophytes in *Lolium perenne* is heavily influenced by host genetics. Front. Plant Sci. 9. doi: 10.3389/fpls.2018.01580 PMC6242978 30483280

[B30] Galindo-González L. Deyholos M. K. (2016). RNA-Seq transcriptome response of flax (*Linum usitatissimum* l.) to the pathogenic fungus *Fusarium oxysporum* f. sp. *lini* . Front. Plant Sci. 7. doi: 10.3389/fpls.2016.01766 PMC5121121 27933082

[B31] Gherbi H. Markmann K. Svistoonoff S. Estevan J. Autran D. Giczey G. . (2008). SymRK defines a common genetic basis for plant root endosymbioses with arbuscular mycorrhiza fungi, rhizobia, and frankia bacteria. Proc. Natl. Acad. Sci. 105, 4928–4932. doi: 10.1073/pnas.0710618105 18316735 PMC2290763

[B32] Giordano W. Hirsch A. M. (2004). The expression of MaEXP1, a *Melilotus alba* expansin gene, is upregulated during the sweetclover-*Sinorhizobium meliloti* interaction. Mol. Plant Microbe Interact. 17, 613–622. doi: 10.1094/MPMI.2004.17.6.613 15195944

[B33] Gundel P. E. Rudgers J. A. Ghersa C. M. (2011). Incorporating the process of vertical transmission into understanding of host–symbiont dynamics. Oikos 120, 1121–1128. doi: 10.1111/j.1600-0706.2011.19299.x

[B34] Haney C. H. Riely B. K. Tricoli D. M. Cook D. R. Ehrhardt D. W. Long S. R. (2011). Symbiotic rhizobia bacteria trigger a change in localization and dynamics of the *Medicago truncatula* receptor kinase LYK3. Plant Cell 23, 2774–2787. doi: 10.1105/tpc.111.086389 21742993 PMC3226205

[B35] Hassing B. Winter D. Becker Y. Mesarich C. H. Eaton C. J. Scott B. (2019). Analysis of *Epichloë festucae* small secreted proteins in the interaction with *Lolium perenne* . PloS One 14, e0209463. doi: 10.1371/journal.pone.0209463 30759164 PMC6374014

[B36] Hettiarachchige I. K. Ekanayake P. N. Mann R. C. Guthridge K. M. Sawbridge T. I. Spangenberg G. C. . (2015). Phylogenomics of asexual *Epichloë* fungal endophytes forming associations with perennial ryegrass. BMC Evol. Biol. 15, 72. doi: 10.1186/s12862-015-0349-6 25902799 PMC4458015

[B37] Hubert D. A. Tornero P. Belkhadir Y. Krishna P. Takahashi A. Shirasu K. . (2003). Cytosolic HSP90 associates with and modulates the *Arabidopsis* RPM1 disease resistance protein. EMBO J. 22, 5679–5689. doi: 10.1093/emboj/cdg547 14592967 PMC275404

[B38] Jelenska J. van Hal J. A. Greenberg J. T. (2010). *Pseudomonas syringae* hijacks plant stress chaperone machinery for virulence. Proc. Natl. Acad. Sci. 107, 13177–13182. doi: 10.1073/pnas.0910943107 20615948 PMC2919979

[B39] Johnson L. J. Bastías D. A. Caradus J. R. Chettri P. Forester N. T. Mace W. J. . (2021). “The dynamic mechanisms underpinning symbiotic epichloë–grass interactions: Implications for sustainable and resilient agriculture,” in Microbiome stimulants for crops (Amsterdam, Netherlands: Woodhead Publishing, Elsevier), 73–108. doi: 10.1016/B978-0-12-822122-8.00008-X

[B40] Johnson L. J. de Bonth A. C. M. Briggs L. R. Caradus J. R. Finch S. C. Fleetwood D. J. . (2013). The exploitation of epichloae endophytes for agricultural benefit. Fungal Divers. 60, 171–88. doi: 10.1007/s13225-013-0239-4

[B41] Johnson L. J. Voisey C. R. Faville M. J. Moon C. D. Simpson W. R. Johnson R. D. . (2019). Advances and perspectives in breeding for improved grass-endophyte associations. Grassland Sci. Europe 24, 351–63. doi: 10.3929/ethz-b-000369264

[B42] Jung H. W. Panigrahi G. K. Jung G. Y. Lee Y. J. Shin K. H. Sahoo A. . (2020). Pathogen-associated molecular pattern-triggered immunity involves proteolytic degradation of core nonsense-mediated mRNA decay factors during the early defense response. Plant Cell 32, 1081–1101. doi: 10.1105/tpc.19.00631 32086363 PMC7145493

[B43] Kadota Y. Shirasu K. (2012). The HSP90 complex of plants. Biochim. Biophys. Acta - Mol. Res. 1823, 689–97. doi: 10.1016/j.bbamcr.2011.09.016 22001401

[B44] Kanehisa M. Goto S. (2000). KEGG: kyoto encyclopedia of genes and genomes. Nucleic Acids Res. 28, 27–30. doi: 10.1093/nar/28.1.27 10592173 PMC102409

[B45] Kanzaki H. Saitoh H. Ito A. Fujisawa S. Kamoun S. Katou S. . (2003). Cytosolic HSP90 and HSP70 are essential components of INF1-mediated hypersensitive response and non-host resistance to *Pseudomonas cichorii* in *Nicotiana benthamiana* . Mol. Plant Pathol. 4, 383–91. doi: 10.1046/j.1364-3703.2003.00186.x 20569398

[B46] Kearse M. Moir R. Wilson A. Stones-Havas S. Cheung M. Sturrock S. . (2012). Geneious basic: an integrated and extendable desktop software platform for the organization and analysis of sequence data. Bioinformatics 28, 1647–49. doi: 10.1093/bioinformatics/bts199 22543367 PMC3371832

[B47] Kim M.-J. Lim G.-H. Kim E.-S. Ko C.-B. Yang K.-Y. Jeong J.-A. . (2007). Abiotic and biotic stress tolerance in *Arabidopsis* overexpressing the multiprotein bridging factor 1a (MBF1a) transcriptional coactivator gene. Biochem. Biophys. Res. Commun. 354, 440–6. doi: 10.1016/j.bbrc.2006.12.212 17234157

[B48] Kombrink A. Sánchez-Vallet A. Thomma B. P. H. J. (2011). The role of chitin detection in plant–pathogen interactions. Microbes Infect. 13, 1168–76. doi: 10.1016/j.micinf.2011.07.010 21856436

[B49] Lewis G. C. Ravel C. Naffaa W. Astier C. Charmet G. (1997). Occurrence of *Acremonium* endophytes in wild populations of *Lolium* spp. in European countries and a relationship between level of infection and climate in France. Ann. Appl. Biol. 130, 227–238. doi: 10.1111/j.1744-7348.1997.tb06828.x

[B50] Liu Y. Burch-Smith T. Schiff M. Feng S. Dinesh-Kumar S. P. (2004). Molecular chaperone Hsp90 associates with resistance protein n and its signaling proteins SGT1 and Rar1 to modulate an innate immune response in plants. J. Biol. Chem. 279, 2101–08. doi: 10.1074/jbc.M310029200 14583611

[B51] Liu J. Elmore J. M. Lin Z.-J. D. Coaker G. (2011). A receptor-like cytoplasmic kinase phosphorylates the host target RIN4, leading to the activation of a plant innate immune receptor. Cell Host Microbe 9, 137–46. doi: 10.1016/j.chom.2011.01.010 21320696 PMC3070605

[B52] Liu J. Nagabhyru P. Schardl C. L. (2017). *Epichloë festucae* endophytic growth in florets, seeds, and seedlings of perennial ryegrass (*Lolium perenne*). Mycologia 109, 691–700. doi: 10.1080/00275514.2017.1400305 29293414

[B53] Liu X. Zhu X. Wang H. Liu T. Cheng J. Jiang H. (2020). Discovery and modification of cytochrome P450 for plant natural products biosynthesis. Synth. Syst. Biotechnol. 5, 187–199. doi: 10.1016/j.synbio.2020.06.008 32637672 PMC7332504

[B54] Lohse M. Nagel A. Herter T. May P. Schroda M. Zrenner R. . (2014). Mercator: a fast and simple web server for genome scale functional annotation of plant sequence data. Plant Cell Environ. 37, 1250–1258. doi: 10.1111/pce.12231 24237261

[B55] Malinovsky F. G. Fangel J. U. Willats W. G. T. (2014). The role of the cell wall in plant immunity. Front. Plant Sci. 5. doi: 10.3389/fpls.2014.00178 PMC4018530 24834069

[B56] Meier S. Ruzvidzo O. Morse M. Donaldson L. Kwezi L. Gehring C. (2010). The *Arabidopsis* wall associated kinase-like 10 gene encodes a functional guanylyl cyclase and is co-expressed with pathogen defense related genes. PloS One 5, e8904. doi: 10.1371/journal.pone.0008904 20126659 PMC2811198

[B57] Morris P. C. (2001). MAP kinase signal transduction pathways in plants. New Phytol. 151, 67–89. doi: 10.1046/j.1469-8137.2001.00167.x 33873387

[B58] Noorifar N. Savoian M. S. Ram A. Lukito Y. Hassing B. Weikert T. W. . (2021). Chitin deacetylases are required for *Epichloë festucae* endophytic cell wall remodeling during establishment of a mutualistic symbiotic interaction with *Lolium perenne* . Mol. Plant Microbe Interact. 34, 1181–1192. doi: 10.1094/MPMI-12-20-0347-R 34058838

[B59] Pauly L. Flajoulot S. Garon J. Julier B. Béguier V. Barre P. (2012). Detection of favorable alleles for plant height and crown rust tolerance in three connected populations of perennial ryegrass (*Lolium perenne* l.). Theor. Appl. Genet. 124, 1139–1153. doi: 10.1007/s00122-011-1775-5 22234605

[B60] Pfeifer M. Martis M. Asp T. Mayer K. F. X. Lübberstedt T. Byrne S. . (2013). The perennial ryegrass GenomeZipper: targeted use of genome resources for comparative grass genomics. Plant Physiol. 161, 571–582. doi: 10.1104/pp.112.207282 23184232 PMC3561004

[B61] Pfender W. F. Slabaugh M. E. (2013). Pathotype-specific QTL for stem rust resistance in *Lolium perenne* . Theor. Appl. Genet. 126, 1213–1225. doi: 10.1007/s00122-013-2048-2 23361523

[B62] Poloni A. Schirawski J. (2014). Red card for pathogens: phytoalexins in sorghum and maize. Molecules 19, 9114–9133. doi: 10.3390/molecules19079114 24983861 PMC6271655

[B63] Quevillon E. Silventoinen V. Pillai S. Harte N. Mulder N. Apweiler R. . (2005). InterProScan: Protein domains identifier. Nucleic Acids Res. 33, W116–W120. doi: 10.1093/nar/gki442 15980438 PMC1160203

[B64] Ralston L. Subramanian S. Matsuno M. Yu O. (2005). Partial reconstruction of flavonoid and isoflavonoid biosynthesis in yeast using soybean type I and type II chalcone isomerases. Plant Physiol. 137, 1375–1388. doi: 10.1104/pp.104.054502 15778463 PMC1088328

[B65] Reinhard K. Matern U. (1989). The biosynthesis of phytoalexins in *Dianthus caryophyllus* l. cell cultures: induction of benzoyl-CoA: anthranilate n-benzoyltransferase activity. Arch. Biochem. Biophys. 275, 295–301. doi: 10.1016/0003-9861(89)90376-7 2817901

[B66] Robinson M. D. McCarthy D. J. Smyth G. K. (2010). edgeR: a bioconductor package for differential expression analysis of digital gene expression data. Bioinformatics 26, 139–140. doi: 10.1093/bioinformatics/btp616 19910308 PMC2796818

[B67] Saikkonen K. Ahlholm J. Helander M. Lehtimäki S. Niemeläinen O. (2000). Endophytic fungi in wild and cultivated grasses in Finland. Ecography 23, 360–366. doi: 10.1111/j.1600-0587.2000.tb00292.x

[B68] Schardl C. L. Leuchtmann A. Chung K.-R. Penny D. Siegel M. R. (1997). Coevolution by common descent of fungal symbionts (*Epichloe* spp.) and grass hosts. Mol. Biol. Evol. 14, 133–143. doi: 10.1093/oxfordjournals.molbev.a025746

[B69] Schmid J. Day R. Zhang N. Dupont P.-Y. Cox M. Schardl C. . (2017). Host tissue environment directs activities of an *Epichloë* endophyte, while it induces systemic hormone and defense responses in its native perennial ryegrass host. Mol. Plant Microbe Interact. 30, 138–149. doi: 10.1094/MPMI-10-16-0215-R 28027026

[B70] Selosse M. Schardl C. L. (2007). Fungal endophytes of grasses: hybrids rescued by vertical transmission? an evolutionary perspective. New Phytol. 173, 452–458. doi: 10.1111/j.1469-8137.2007.01978.x 17244040

[B71] Sels J. Mathys J. de Coninck B. M. A. Cammue B. P. A. de Bolle M. F. C. (2008). Plant pathogenesis-related (PR) proteins: A focus on PR peptides. Plant Physiol. Biochem. 46, 941–950. doi: 10.1016/j.plaphy.2008.06.011 18674922

[B72] Supek F. Bošnjak M. Škunca N. Šmuc T. (2011). REVIGO summarizes and visualizes long lists of gene ontology terms. PloS One 6, e21800. doi: 10.1371/journal.pone.0021800 21789182 PMC3138752

[B73] Takahashi S. Yeo Y.-S. Zhao Y. O’Maille P. E. Greenhagen B. T. Noel J. P. . (2007). Functional characterization of premnaspirodiene oxygenase, a cytochrome P450 catalyzing regio-and stereo-specific hydroxylations of diverse sesquiterpene substrates. J. Biol. Chem. 282, 31744–31754. doi: 10.1074/jbc.M703378200 17715131 PMC2695360

[B74] Tan Y. Y. Spiering M. J. Scott V. Lane G. A. Christensen M. J. Schmid J. (2001). In planta regulation of extension of an endophytic fungus and maintenance of high metabolic rates in its mycelium in the absence of apical extension. Appl. Environ. Microbiol. 67, 5377–5383. doi: 10.1128/AEM.67.12.5377-5383.2001 11722882 PMC93319

[B75] Thoms D. Liang Y. Haney C. H. (2021). Maintaining symbiotic homeostasis: how do plants engage with beneficial microorganisms while at the same time restricting pathogens? Mol. Plant Microbe Interact. 34, 462–469. doi: 10.1094/MPMI-11-20-0318-FI 33534602

[B76] Thorvaldsdóttir H. Robinson J. T. Mesirov J. P. (2013). Integrative genomics viewer (IGV): High-performance genomics data visualization and exploration. Brief. Bioinf. 14, 178–192. doi: 10.1093/bib/bbs017 PMC3603213 22517427

[B77] Tian T. Liu Y. Yan H. You Q. Yi X. Du Z. . (2017). agriGO v2.0: a GO analysis toolkit for the agricultural communit Update. Nucleic Acids Res. 45, W122–W129. doi: 10.1093/nar/gkx382 28472432 PMC5793732

[B78] Trusov Y. Botella J. R. (2016). Plant G-proteins come of age: breaking the bond with animal models. Front. Chem. 4. doi: 10.3389/fchem.2016.00024 PMC4877378 27252940

[B79] van Ooijen G. Lukasik E. van den Burg H. A. Vossen J. H. Cornelissen B. J. C. Takken F. L. W. (2010). The small heat shock protein 20 RSI2 interacts with and is required for stability and function of tomato resistance protein I-2. Plant J. 63, 563–572. doi: 10.1111/j.1365-313X.2010.04260.x 20497382 PMC2988412

[B80] Verhoeven K. J. F. Simonsen K. L. McIntyre L. M. (2005). Implementing false discovery rate control: Increasing your power. Oikos 108, 643–647. doi: 10.1111/j.0030-1299.2005.13727.x

[B81] Wang R. Luo S. Clarke B. B. Belanger F. C. (2021). The *Epichloë festucae* antifungal protein efe-AfpA is also a possible effector protein required for the interaction of the fungus with its host grass *Festuca rubra* subsp. *rubra* . Microorganisms 9, 140. doi: 10.3390/microorganisms9010140 33435432 PMC7827515

[B82] Wang G. Wang G. Zhang X. Wang F. Song R. (2012). Isolation of high quality RNA from cereal seeds containing high levels of starch. Phytochem. Anal. 23, 159–163. doi: 10.1002/pca.1337 21739496

[B83] White J. F. Jr. Breen J. P. Morgan-Jones G. (1991). Substrate utilization in selected *Acremonium*, *Atkinsonella* and *Balansia* species. Mycologia 83, 601–610. doi: 10.1080/00275514.1991.12026059

[B84] Wu T. D. Reeder J. Lawrence M. Becker G. Brauer M. J. (2016). GMAP and GSNAP for genomic sequence alignment: enhancements to speed, accuracy, and functionality. Methods Mol. Biol. 1418, 283–334. doi: 10.1007/978-1-4939-3578-9_15 27008021

[B85] Zadoks J. C. Chang T. T. Konzak C. F. (1974). A decimal code for the growth stages of cereals. Weed Res. 14, 415–421. doi: 10.1111/j.1365-3180.1974.tb01084.x

[B86] Zhang W. Card S. D. Mace W. J. Christensen M. J. McGill C. R. Matthew C. (2017). Defining the pathways of symbiotic *Epichloë* colonization in grass embryos with confocal microscopy. Mycologia 109, 153–161. doi: 10.1080/00275514.2016.1277469 28402784

[B87] Zhang Z. Ersoz E. Lai C.-Q. Todhunter R. J. Tiwari H. K. Gore M. A. . (2010). Mixed linear model approach adapted for genome-wide association studies. Nat. Genet. 42, 355–360. doi: 10.1038/ng.546 20208535 PMC2931336

