# Supplementary material for: Epichloë seed transmission efficiency is influenced by plant defense response mechanisms
Source: Front Plant Sci. 2022 Oct 21;13:1025698. doi: 10.3389/fpls.2022.1025698 (PMC9635450; doi:10.3389/fpls.2022.1025698)
Supplement: Supplementary file 5 [file Presentation_1.pptx]

## Slide 1
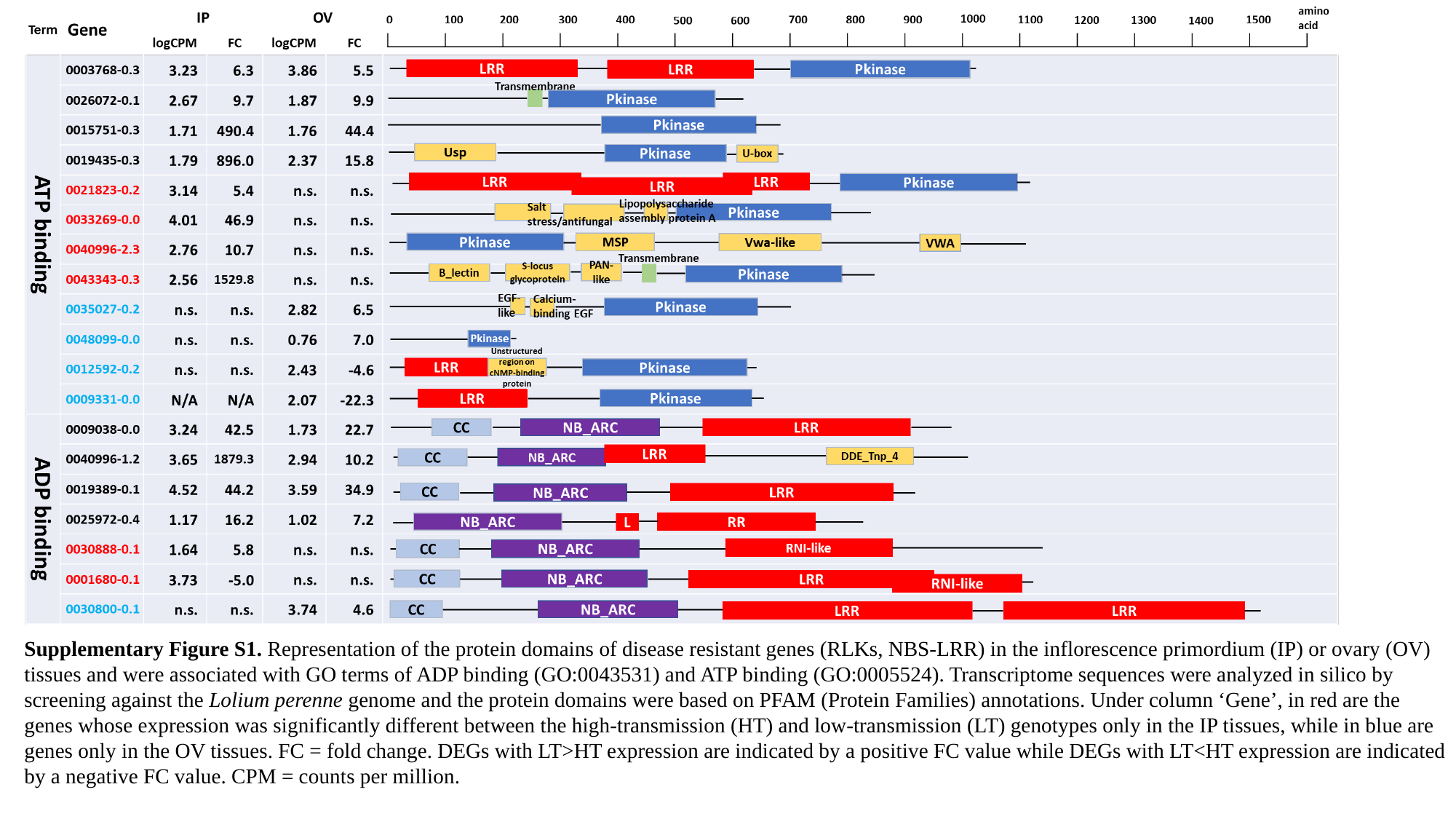

Supplementary Figure S1. Representation of the protein domains of disease resistant genes (RLKs, NBS-LRR) in the inflorescence primordium (IP) or ovary (OV) tissues and were associated with GO terms of ADP binding (GO:0043531) and ATP binding (GO:0005524). Transcriptome sequences were analyzed in silico by screening against the Lolium perenne genome and the protein domains were based on PFAM (Protein Families) annotations. Under column ‘Gene’, in red are the genes whose expression was significantly different between the high-transmission (HT) and low-transmission (LT) genotypes only in the IP tissues, while in blue are genes only in the OV tissues. FC = fold change. DEGs with LT>HT expression are indicated by a positive FC value while DEGs with LT<HT expression are indicated by a negative FC value. CPM = counts per million.
